# Gypenoside Inhibits Endothelial Cell Apoptosis in Atherosclerosis by Modulating Mitochondria through PI3K/Akt/Bad Pathway

**DOI:** 10.1155/2020/2819658

**Published:** 2020-06-20

**Authors:** Nan Song, Lianqun Jia, Huimin Cao, Yixin Ma, Ning Chen, Si Chen, Xiaoming Lv, Guanlin Yang

**Affiliations:** ^1^Key Laboratory of Ministry of Education for TCM Viscera-State Theory and Applications, Liaoning University of Traditional Chinese Medicine, Shenyang 110847, China; ^2^National Local Joint Engineering Laboratory for the Prevention and Treatment of Cardioencephalopathy with Integrated Traditional Chinese and Western Medicine, Shenyang 110847, China

## Abstract

Atherosclerosis remains the most common cause of deaths worldwide. Endothelial cell apoptosis is an important process in the progress of atherosclerosis, as it can cause the endothelium to lose their capability in regulating the lipid homeostasis, inflammation, and immunity. Endothelial cell injury can disrupt the integrity and barrier function of an endothelium and facilitate lipid deposition, leading to atherogenesis. Chinese medicine techniques for preventing and treating atherosclerosis are gaining attention, especially natural products. In this study, we demonstrated that gypenoside could decrease the levels of serum lipid, alleviate the formation of atherosclerotic plaque, and lessen aortic intima thickening. Gypenoside potentially activates the PI3K/Akt/Bad signal pathway to modulate the apoptosis-related protein expression in the aorta. Moreover, gypenoside downregulated mitochondrial fission and fusion proteins, mitochondrial energy-related proteins in the mouse aorta. In conclusion, this study demonstrated a new function of gypenoside in endothelial apoptosis and suggested a therapeutic potential of gypenoside in atherosclerosis associated with apoptosis by modulating mitochondrial function through the PI3K/Akt/Bad pathway.

## 1. Introduction

Atherosclerosis is one of the important causes of death worldwide despite drug therapy and surgical operation treatment [1]. The disease causes arteries to change and is characterized as fatty plaque formation [2]. These plaques may crack, possibly leading to vessel occlusion and clinical syndrome such as cardiopathy and strokes [3]. As atherosclerosis has a high morbidity and mortality, it is urgent to discover its disease mechanism and find new therapeutic targets.

Gynostemma pentaphyllum is the root or whole plant of Gynura Gynostemma [4]. Some studies have identified that Gynostemma pentaphyllum has functions in lowering blood fat, preventing arteriosclerosis, resisting oxidation, lowering blood sugar, and regulating immunity [5–9]. Gypenosides is an extraction product of Gynostemma pentaphyllum, which has multiple pharmacological activities [10–12]. Ge et al. found gypenosides could secure cardiac muscles and improve the function in diabetic cardiomyopathy rats [13]. Moreover, gypenosides were demonstrated to alleviate myocardial ischemia-reperfusion injury through reduction of oxidative stress and protection of mitochondrial function [14]. Yang et al. reported that Gypenoside XVII could prevent atherosclerosis through decreasing endothelia apoptosis and oxidative stress [15]. However, the mechanism by which gypenosides and their components inhibit endothelial cell apoptosis and prevent atherosclerosis is not fully understood.

The apolipoprotein E knock out (ApoE−/−) mouse is one of excellent models of the mimicking human atherosclerosis, which shows the spontaneously occurring lesions distributed in the vasculature [16]. Endothelial cell injury apoptosis plays an important role in the development of atherosclerosis, possibly causing the endothelium not to regulate lipid homeostasis, inflammation, and immunity [17, 18]. Endothelial cell injury can interrupt the endothelial integrity and defense function and promote lipid deposition, leading to atherogenesis [19, 20]. The detailed mechanisms were the development of apoptosis, which was regulated by the mitochondria and its related proteins. And both conditions could lead to cell death. Cytochrome C was released to the cytosol, as the mitochondrial membrane permeability was changed by extracellular or intracellular signals, resulting to create an apoptotic signal. [21]. Cytochrome C could recruit Apaf-1 and procaspase 9 to trigger the apoptosome, an upstream factor of the caspase 9/3 signaling cascade, the canonical pathway for apoptotic cell death [22].

Our study demonstrates that phosphoinositide-3 kinase (PI3K) transduces survival effects, which depends on the Akt kinase phosphorylation and activation and then the proapoptotic Blc-2 family protein Bad phosphorylation and inhibition. PI3K plays an important function in growth factor signal transduction. Under various cytokines and physiochemical factor activation, PI3K could produce myoinositol as a second messenger [23]. Akt also performs crucial roles in many biological processes including cell metabolism, cell cycle, cell growth, and apoptosis [24–26]. Akt can be activated by various growth and survival factors. PI3K activation mediates the phosphorylation and partial or complete activation of Akt. The activation of Akt could inhibit phosphorylation of apoptotic signaling proteins or regulate the transcription factors to modulate apoptosis. Akt was reported to be involved in phosphorylation and inactivation of Bad and inhibition of cell death [27]. Several Bcl-2 family proteins such as Bcl-2 and Bcl-xL promote cell survival; meanwhile, other proteins such as Bad and Bax could increase the cell death [28]. It has also been shown that Bcl-2 family members, located on the mitochondrial membrane, could change mitochondrial membrane permeability and lead to apoptosis [29, 30]. PI3K is an important catalytic enzyme that regulates the production of lipid derivatives with second messenger characteristics, directly affecting atherosclerosis development [31, 32]. Our study focused on the exploration of function and underlying mechanisms of gypenoside in the apoptosis of endothelial cell in atherosclerosis.

## 2. Materials and Methods

### 2.1. Animals and Group

Thirty ApoE−/− mice (male, 8 weeks, 18–20 g weight) with C57BL/6J background and ten C57BL mice were acclimated before use in experiments. Tap water and chow were provided ad libitum. All animal experiments followed the guidelines of the Animal Committee of Liaoning University of Traditional Chinese Medicine, China.

### 2.2. Materials and Reagents

Gypenoside was obtained from Xi'an Realin Biotechnology Co., Ltd., China. TC, TG, LDL-C, and HDL-C Test Kits were obtained from Sichuan Maker Biotechnology Co. Ltd., China. The real-time PCR Kits were obtained from TaKaRa Biotechnology Co. Ltd., Dalian, China. The BCA Protein Assay Kit, RIPA Lysis Buffer, and SDS-PAGE Gel Preparation Kits were obtained from Beijing SOLARBIO Technology Co., Ltd. Dulbecco's modified Eagle's medium (high glucose) and phosphate-buffered saline (PBS) were obtained from HyClone (Logan, UT, USA). Fetal bovine serum (FBS), penicillin-streptomycin solution, and 0.25% Trypsin-EDTA were obtained from HyClone, UT, USA.

### 2.3. Experiment Grouping Design In Vivo

Three groups of mice were used in testing including C57BL/6L mice (normal group, *n* = 10), ApoE−/− mice (ApoE−/− group, *n* = 10) under high-fat diet treatment of six weeks, and ApoE−/− mice treated with gypenoside 2.973 mg/kg/d gypenoside (gypenoside group, *n* = 10) or simvastatin (simvastatin group, *n* = 10) for an additional 7 weeks. All ApoE−/− mice were fed a high-fat diet containing 21% (wt/wt) fat from lard supplemented with 0.15% (wt/wt) cholesterol for 13weeks. Ten C57BL mice were fed with standard chow diet containing 4% fat, which were grouped in the normal group. All mice were inspected once per day. Drugs were dissolved in water. The water consumption was monitored twice weekly, and drug concentration was adjusted as required.

### 2.4. Experiment Grouping Design In Vitro

EA.hy926 cells were purchased from the Cell Bank of the Chinese Academy of Sciences (Shanghai, China). EA.hy926 cells were cultured in Dulbecco's modified Eagle's medium (DMEM, HyClone, Logan, UT, USA) supplemented with 10% fetal bovine serum (FBS, HyClone, Logan, UT, USA) and 100 U/ml penicillin and 100 mg/ml streptomycin (Sigma-Aldrich Co., St. Louis, MO, USA). Cells were cultivated in an incubator at 37°C, 5% CO2, and the medium was changed every 2-3 days. Cells were randomly divided into 9 experimental groups as follows: control group; LDL group [cells treated with 100 *μ*g/ml oxidized low-density lipoprotein (ox-LDL, Yiyuan Biotechnology) for 24 h]; Gypenoside (Gps) group, Gypenoside XILX (GpXILX) group, and Ginsenoside Rb3 (Rb3) group, in which 100 *μ*g/ml Gps, GpXILX, or Rb3, respectively, and 100 *μ*g/ml ox-LDL were added to the cells for 24 h. In the Modle+LY group, Gps+LY group, GpXILX+LY group, and Rb3+LY group, 10 *μ*M LY294002 was added and then 100 *μ*g/ml Gps, GpXILX, Rb3, and 100 *μ*g/ml ox-LDL were added to the medium after 2 h.

### 2.5. Detection of Serum Lipid Profile

The kits (Sichuan Maker Biotechnology, China) were used to assess serum levels of total cholesterol (TC), Triglyceride (TG), low-density lipoprotein cholesterol (LDL-C), and high-density lipoprotein cholesterol (HDL-C) according to previously described methods [33] by an automatic biochemical analyzer (Toshiba, Japan).

### 2.6. Histopathological Analysis

The entire aorta of mice was rapidly cut out and immersed in 10% neutral buffered formalin (pH 7.4) for fixation. Then arterial tissues were embedded in paraffin, cut transversely in 5 mm thick, and stained with H&E.

### 2.7. PCR Array Analysis

The mitochondrial energy metabolism signaling pathway was analyzed in the mouse aortic root by the RT2 Profiler™ PCR Array System (Qiagen). 40 *μ*l cDNA was mixed with 2x SABiosciences RT2 qPCR Master Mix (Qiagen), followed by adding sterile water to a total volume of 2700 *μ*l. 25 *μ*l mixture was added to each well of the PCR array plate. PCR amplification and fluorescence detection was performed using the TaqMan Gene Expression Master Mix on ABI-7500 (Applied Biosystems) in a total volume of 20 *μ*l. Thermal cycling involved 95°C for 10 min, then 95°C for 15 s, 55°C for 40 s, with 40 cycles, and 72°C for 30 s. The gene expression was determined using the *ΔΔ*CT method with GAPDH as an internal control.

### 2.8. Real-Time Quantitative PCR

Aortic roots were removed from mice from all groups and stored at −80°C to examine RNA levels of Atp12a, Cox5a, Ndufb6, and Sdhc. Total RNA was extracted from aortas by the TRIzol kit. Primers for Atp12a, Cox5a, Ndufb6, and Sdhc are shown in Table 1. The protocol of RT-PCR was according to the previously described method [34]. The model of the qPCR machine was an ABI 7500 (America). Data was analyzed by the 2^−*ΔΔ*CT^ method.

### 2.9. Western Blot

Total proteins were extracted from cells or tissues using RIPA Lysis Buffer. Protein concentration was measured by BCA Protein Assay Kit. To examine the expression of proteins, the same amount of total proteins was loaded on an 8-12% sodium dodecyl sulfate-polyacrylamide gel electrophoresis (SDS-PAGE) gel. Proteins were transferred into PVDF membranes. After being blocked in skim milk solution, the membrane was incubated overnight separately with antibodies anti-*β*-actin (Santa Cruz Biotechnology, Santa Cruz, CA, USA), anti-PI3K, anti-p-Akt, anti-p-Bad, anti-Cyt-c, anti-cleaved caspase 9, anti-cleaved caspase 3, anti-PARP, anti-DRP1, and anti-Mfn2 (Cell Signal, CST, USA). After that, the membrane was incubated with the secondary HRP-conjugated goat anti-rabbit antibodies (Santa Cruz Biotechnology). Proteins were visualized using an enhanced chemiluminescence kit from Thermo Fisher Scientific (Massachusetts, USA). ImageJ software (Alpha View SA) was used to perform densitometric analysis.

### 2.10. Immunofluorescence Staining

After the arterial tissues were embedded in paraffin and cut into 5 mm thick sections, the sections were incubated in PBS containing 10% normal goat serum, 3% (*w*/*v*) bovine serum albumin, and 0.05% Tween-20 for 2 h at room temperature and incubated overnight at 4°C with the following primary antibodies: mouse anti-Mfn2 (1 : 50; Abcam) and rabbit anti-DRP1 (1 : 100; Cell Signaling Technology). Finally, the sections were incubated with FITC-TSA- and CY3-TSA-conjugated secondary antibodies (Servicebio, Wuhan, China) for 2 h at 4°C. The results were observed using a Leica fluorescence microscope.

### 2.11. Caspase 3 Activity Detection

Renal caspase 3 activity was detected by fluorescent caspase-specific substrates Ac-DEVD-7-*p*NA (Beyotime, China). Briefly, 10 mg proteins were loaded by the reaction buffer and cultivated at 37°C for 2 h. The enzyme-catalyzed release of AFC was quantified in a fluorimeter at 405 nm.

### 2.12. Statistical Analysis

Data were showed as the mean and standard deviation (SD). The differences among the groups were analyzed by ANOVA using GraphPad Prism 8 (San Diego, CA, USA). *P* < 0.05 was considered to be statistically significant.

## 3. Results

### 3.1. Effects of Gypenoside on Serum Lipid Levels

**Figure 1:**
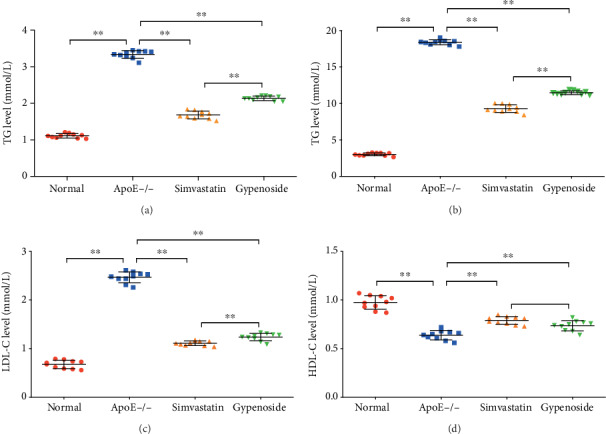
Gypenoside decreases serum lipid levels. The serum lipid profile (TC, TG, LDL-C, and HDL-C levels) was analyzed in the normal, ApoE−/−, gypenoside, and simvastatin groups. (a) TG level, (b) TC level, (c) LDL-C level, and (d) HDL-C level. All results are expressed as the mean ± SD of four different experiments (*n* = 10). ^∗∗^*P* < 0.01 between each group.

Lipid profiles in serum from several groups of mice are shown in Figures 1(a)–1(d). The mice of the ApoE−/− group showed higher serum levels.

The serum levels of TG, TC and LDL-C were significantly higher in the ApoE−/− group than those in the normal group; the level of HDL-C was lower in the ApoE−/− group than that in the normal group (*P* < 0.01). Treatment with gypenoside and simvastatin significantly decreased the levels of TC, TGs, and LDL-C compared to the ApoE−/− group (*P* < 0.01, Figures 1(a)–1(c)). Protective HDL-C was increased (*P* < 0.01) under gypenoside and simvastatin treatment (Figure 1(d)). We noted that gypenoside consistently decreased TG, TC, and LDL-C more than simvastatin treatment (*P* < 0.01, Figures 1(a)–1(c)).

### 3.2. Change of Atherosclerotic Plaques under Gypenoside Treatment

**Figure 2:**
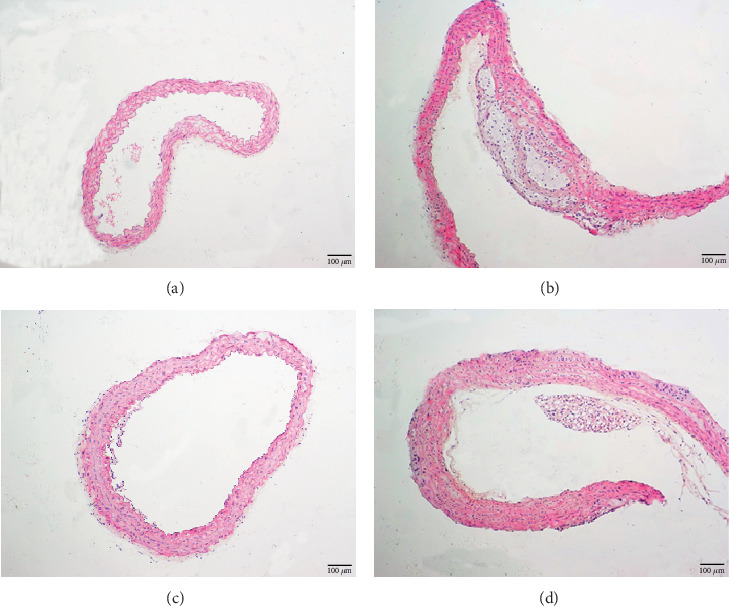
Pathomorphological changes of the aorta. Morphological observations of aortas by H&E staining. (a) Normal group, (b) ApoE−/− group, (c) simvastatin group, and (d) gypenoside group.

H&E staining presented normal walls of the aorta with intact uninterrupted endothelial lining, a smooth and uninterrupted aortic intima, and a clear demarcation of the aortic tunica media and aortic adventitia in the normal group (Figure 2(a)). ApoE−/− mice were characterized by decreased aortic intima smooth muscle cells; the presence of many plaques, infiltrating lymphocytes, neutrophils, and foam cells; and a large number of cytoplasmic vacuoles (Figure 2(b)). In the gypenoside and simvastatin groups, the atherosclerotic plaques and thickening of the aortic intima were alleviated (Figures 2(c) and 2(d)).

### 3.3. Gypenoside Potentially Activates PI3K/Akt/Bad Pathway to Regulate the Apoptosis-Related Proteins in the Aorta

**Figure 3:**
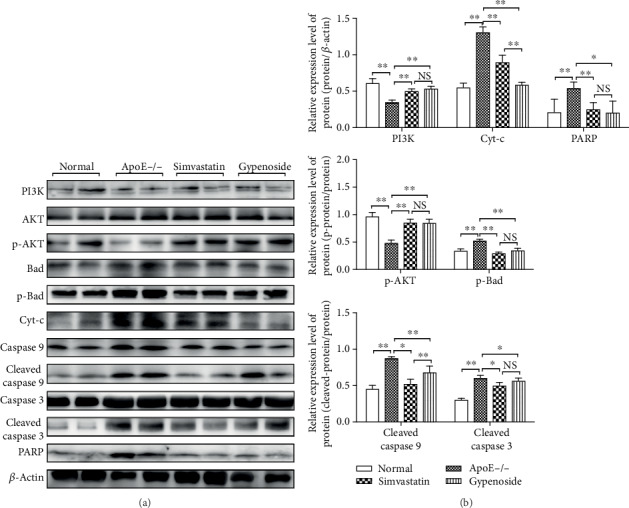
Gypenoside modulates the expression of apoptosis pathway proteins. Gypenoside effectively regulated the expression of apoptotic proteins in the PI3K/Akt/Bad pathway. Gypenoside enhanced the expression of PI3K and p-Akt and markedly downregulated the expression of p-Bad, Cyt-c, cleaved caspase 9, cleaved caspase 3, and PARP. Bar chart results are expressed as the mean ± SD of four different experiments (*n* = 3). (a) Western blot; (b) bar charts; ^∗^*P* < 0.05 and ^∗∗^*P* < 0.01 between each group.

PI3K, p-Akt, and p-Bad were significantly downregulated in the mice of the ApoE−/− group compared to those in the normal group (*P* < 0.01). Treatment with simvastatin and gypenoside significantly upregulated (*P* < 0.01) PI3K p-Akt and p-Bad protein levels. Furthermore, Cyt-c, cleaved caspase 9, cleaved caspase 3, and PARP were significantly upregulated in the ApoE−/− group compared to the normal group (*P* < 0.01). Treatment with simvastatin and gypenoside significantly downregulated the expression of Cyt-c, cleaved caspase 9, cleaved caspase 3, and PARP (*P* < 0.01 or *P* < 0.05, Figure 3).

### 3.4. Gypenoside Decreases Bioactivity of Caspase 3

**Figure 4:**
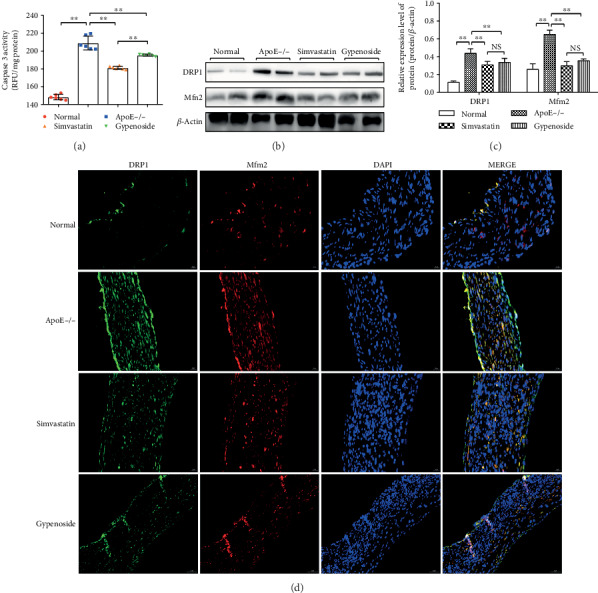
Gypenoside decreases caspase 3 activity and the expression of mitochondrial fission and fusion proteins in the aorta. (a) Caspase 3 activity, as analyzed by the corresponding kit in the normal, ApoE−/−, gypenoside, and simvastatin groups. Results are expressed as the mean ± SD of four different experiments (*n* = 6). (b, c) Western blot result of DRP1 and MFN2 expression in the aorta; bar chart results are expressed as the mean ± SD of four different experiments (*n* = 3); ^∗^*P* < 0.05 and ^∗∗^*P* < 0.01 between each group. (d) Immunofluorescence results of DRP1 and MFN2 expression in the aorta.

Caspases is related with the final stages of apoptosis. The activation of effector caspase 3 is an important indicator of apoptosis. Caspase 3 was showed to be significantly activated in the ApoE−/− group compared with that in normal group (*P* < 0.01). Treatment with gypenoside and simvastatin significantly inhibited the caspase 3 activity compared to the ApoE−/− group (*P* < 0.01, Figure 4(a)).

### 3.5. Gypenoside Decreases the Mitochondrial Fission and Fusion Proteins of the Aorta

DRP1 and Mfn2 protein expressions were increased in ApoE−/− mice compared to those in the normal group (*P* < 0.01). Treatment with simvastatin and gypenoside significantly downregulated (*P* < 0.01) the levers of DRP1 and Mfn2 protein detected by western blot (Figures 4(b) and 4(c)) and immunofluorescence assay (Figure 4(d).

### 3.6. Mitochondrial Energy-Related Gene Expression in the Mouse Aorta

We examined the expression of 84 genes in the mitochondrial energy metabolism signaling pathway in the aortic root of normal and ApoE−/− mice by Mouse Mitochondrial Energy Metabolism PCR Array. 25 genes were significantly upregulated (fold change > 2), and 7 genes were downregulated (fold change < 0.5). We chose mitochondrial energy-related genes by screening across four specimen replication experiments (Table 2).

### 3.7. Gypenoside Regulates the Expression of Atp12a, Cox5a, Ndufb6, and Sdhc mRNA in the Aorta

**Figure 5:**
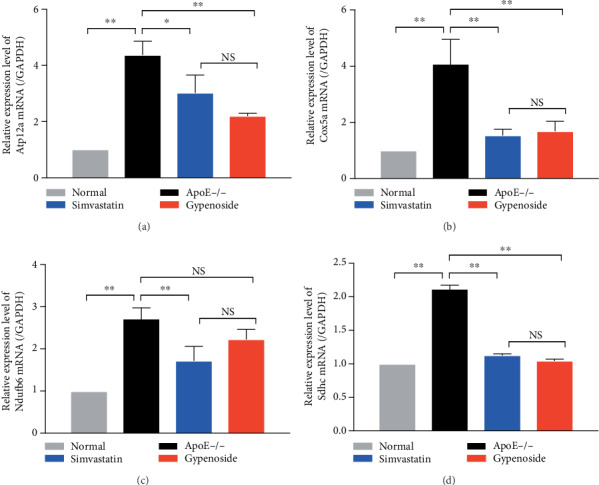
Gypenoside regulates mRNA expression of mitochondrial energy-related genes in the aorta. Mitochondrial energy-related genes Atp12a, Cox5a, Ndufb6, and Sdhc were analyzed by qPCR. (a) Atp12a mRNA is increased in ApoE−/− mice and reduced by gypenoside and simvastatin treatment; (b) Cox5a mRNA is increased in ApoE−/− mice and reduced by gypenoside and simvastatin treatment; (c) Ndufb6 mRNA is increased in ApoE−/− mice and is only reduced by simvastatin treatment; (d) Sdhc mRNA is increased in ApoE−/− mice and reduced by gypenoside and simvastatin treatment. All results are expressed as the mean ± SD of four different experiments (*n* = 3). ^∗∗^*P* < 0.01 between each group.

We performed qPCR analyses to investigate whether gypenoside affected the transcriptional regulation of the mitochondrial energy-related genes implicated above. As shown in Figure 5, Atp12a, Cox5a, Ndufb6, and Sdhc mRNA were significantly increased in ApoE−/− mice compared with those in normal mice (*P* < 0.01). The expressions of Atp12a, Cox5a, and Sdhc mRNA were significantly downregulated under simvastatin and gypenoside treatment (*P* < 0.01 or *P* < 0.05, Figure 5).

### 3.8. Gypenosides, Gypenoside XILX, and Ginsenoside Rb3 Prevent ox-LDL-Induced Apoptosis through PI3K/Akt/Bad Pathway In Vitro

**Figure 6:**
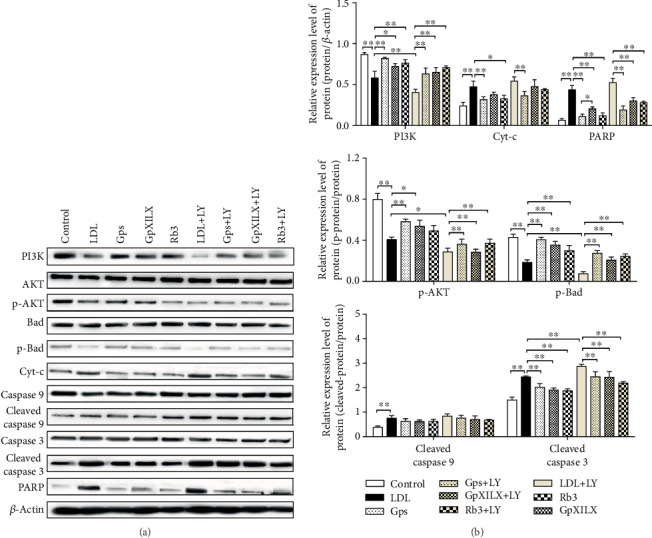
Gypenosides, Gypenoside XILX, and Ginsenoside Rb3 modulate expression of PI3K/Akt/Bad pathway proteins and prevent expression of apoptosis proteins in ox-LDL-induced EA.hy926 cells. Gypenosides (Gps), Gypenoside XILX (GpXILX), and Ginsenoside Rb3 (Rb3) effectively regulated expression of PI3K/Akt/Bad pathway proteins. Gps, GpXILX, and Rb3 enhanced expression of PI3K, p-Akt, and p-Bad. All results are expressed as the mean ± SD of four different experiments (*n* = 3). ^∗^*P* < 0.05 and ^∗∗^*P* < 0.01 between each group.

We investigated the relationship of the PI3K/Akt/Bad pathway and Gypenosides (Gps), Gypenoside XILX (GpXILX), and Ginsenoside Rb3 (Rb3) in preventing ox-LDL-induced apoptosis in EA.hy926 cells. ox-LDL treatment could significantly inactivate the phosphorylation of Akt at Ser473 and Bad at Ser136. The apoptotic proteins including Cyt-c, cleaved caspase 9, caspase 3, and PARP were also increased in ox-LDL-treated cells. However, these effects were reversed by Gps, GpXILX, and Rb3. The effect of Gps, GpXILX, and Rb3 was abrogated as cells were treated with the PI3K inhibitor, LY294002 (LY). There results indicate that Gps, GpXILX, and Rb3 prevent ox-LDL-induced apoptosis in vitro through the PI3K/Akt/Bad signal pathway (Figure 6).

### 3.9. Gps, GpXILX, and Rb3 Modulate the Mitochondrial Fission and Fusion Proteins through the PI3K/Akt/Bad Pathway

**Figure 7:**
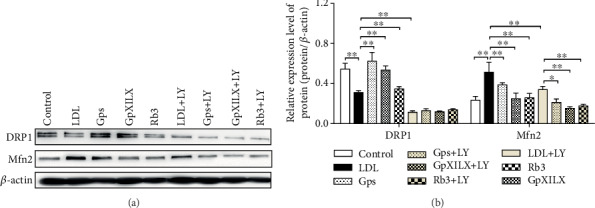
Gypenosides, Gypenoside XILX, and Ginsenoside Rb3 modulate the expression of mitochondrial fission and fusion proteins in ox-LDL-induced EA.hy926 cells. Gypenosides (Gps), Gypenoside XILX (GpXILX), and Ginsenoside Rb3 (Rb3) effectively regulated expression of mitochondrial fission and fusion proteins; Gps markedly downregulated the expression of DRP1 and Mfn2. The results are expressed as the mean ± SD of four different experiments (*n* = 3). (a) Western blot; (b) bar charts; ^∗^*P* < 0.05 and ^∗∗^*P* < 0.01 between each group.

Gps, GpXILX, and Rb3 modulated mitochondrial fission and fusion protein expression in EA.hy926 cells induced by ox-LDL and prevent ox-LDL-induced apoptosis through the PI3K/Akt/Bad pathway. Treatment with ox-LDL significantly reduced DRP1 protein expression and increased Mfn2 compared with controls. And these functions could be blocked under treatment of Gps, GpXILX, and Rb3. The effects of Gps, GpXILX, and Rb3 were abrogated following treatment with LY. These results indicate that Gps, GpXILX, and Rb3 prevent ox-LDL-induced expression of DRP1 and Mfn2 protein through the PI3K/Akt/Bad pathway in EA.hy926 cells (Figure 7).

### 3.10. Gps, GpXILX, and Rb3 Promote the Bioactivity of Mitochondrial Respiratory Chain Complex Enzymes in ox-LDL-Induced EA.hy926 Cells

**Figure 8:**
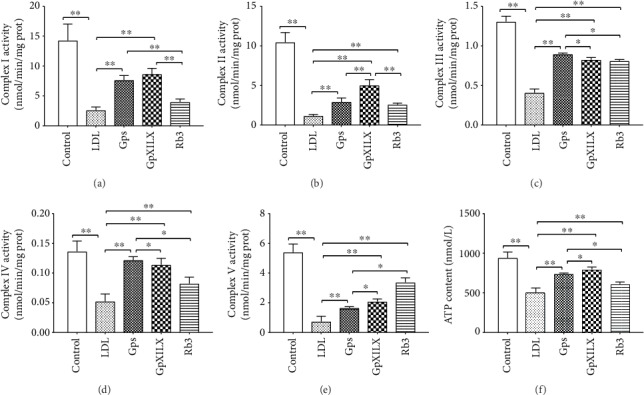
Effect of Gypenosides, Gypenoside XILX, and Ginsenoside Rb3 on mitochondrial respiratory chain complex enzymes I, II, III, IV, and V and ATP content. (a–e) Gps, GpXILX, and Rb3 effectively increase the activity of mitochondrial respiratory chain complex enzymes I, II, III, IV, and V. All results are expressed as the mean ± SD of five different experiments (*n* = 6); (f) ATP content was measured in EA.hy926 cells. LDL group cells exhibited a decrease in ATP content, which was partially rescued by Gps, GpXILX, and Rb3. All results are expressed as the mean ± SD of five different experiments (*n* = 3). ^∗^*P* < 0.05 and ^∗∗^*P* < 0.01 between each group.

The activities of mitochondrial respiratory chain complex enzymes I, II, III, IV, and V were determined by the corresponding kits. In the LDL group, the activity of detected enzymes was reduced. However, Gps, GpXILX, and Rb3 significantly enhanced the activity of the five mitochondrial respiratory chain complex enzymes compared with those in the LDL group (Figures 8(a)–8(e)).

### 3.11. Gps, GpXILX, and Rb3 Increase ATP Content in ox-LDL-Induced EA.hy926 Cells

The ATP content was showed to decrease significantly in ox-LDL-induced EA.hy926 cells of the LDL group compared with those the control group (*P* < 0.01). The treatment of Gps, GpXILX, and Rb3 significantly increased the level of ATP (*P* < 0.01, Figure 8(f)).

## 4. Discussion

Gypenosides are from the main components of Gynostemma pentaphyllum, which have been proved to be effective in the therapy of cardiovascular diseases, especially atherosclerosis [35]. Previous results showed that gypenoside treatment could decrease the levels of TC, TGs, and LDL-C and gypenoside promoted the level of protective HDL-C significantly to be above its normal level. Gypenoside-treated ApoE−/− mice also had alleviated atherosclerotic plaque formation and aortic intima thickening. However, the mechanism by which gypenoside prevents atherosclerosis remained unknown. Here, we studied the function of gypenoside on atherosclerosis and the relationship with the PI3K/Akt/Bad apoptotic pathway.

Mitochondria are a major cellular organelle involved in cell growth, differentiation, message transmission, apoptosis, and energy supply [36]. 95% of the energy required for cell survival is provided by the mitochondrial respiratory chain [37]. Cyt-c is a primary component of mitochondrial respiratory chain [38]. Cyt-c, released from mitochondria, could cause breaks in the electron transport chain, production of oxygen free radicals, and decreased ATP production, eventually leading to apoptosis [39, 40].

Apoptosis is known as the process of programmed cell death with important roles in regulating cellular homeostasis across the body. Apoptosis levels in vascular endothelial cells closely relate to the formation and development of atherosclerosis [18]. The endogenous mitochondrial pathway is one of the major apoptotic pathways [41, 42]. Cyt-c, released from mitochondrial to the cytoplasm, could activate caspase 3 and caspase 9, which is a key step for inducing apoptosis. When induced by Ala-Pro-Phe-chloromethylketone (APF) and other factors in the presence of ATP/dATP, Cyt-c can bind Apaf-1 and caspase 9 to form an apoptotic complex [43]. Caspase 9 is then activated, allowing it to activate caspase 3 to further continue the caspase activation cascades [44]. Activated caspase 3 could cleave the DNA repair enzyme PARP into small fragments, blocking its normal function and leading to DNA cleavage, eventually causing apoptosis [45]. Our results showed that gypenoside treatment significantly decreases caspase 3 activity (*P* < 0.01) in the ApoE−/− group. Mitochondrial apoptosis-related proteins had significantly increased expression in ApoE−/− mouse arterial endothelial cells, and the mitochondrial energy-related genes Atp12a and Cox5a were found expressed in these cells. Ndufb6 and SDHC mRNA were also significantly increased in ApoE−/− cells, but this was blocked by intervention with gypenosides. Our results show that gypenosides can inhibit the development of atherosclerosis in ApoE-deficient mice via regulating changes in mitochondrial apoptosis and energy.

The PI3K/Akt/Bad signaling pathway performs an important function in inhibiting mitochondria-mediated apoptosis [46]. Based on the close relationship between the PI3K/Akt/Bad pathway and apoptosis, we studied the effect of PI3K in our system. PI3K is a phosphatidylinositol kinase with activities as a serine/threonine-specific protein kinase and a phosphatidylinositol kinase [47, 48]. After activation, phosphatidylinositol family members on the cell membrane can be phosphorylated and the downstream signal molecule Akt can be recruited and activated. Then activated Akt phosphorylates Ser136/Ser112 residues of the Bad protein [49]. Phosphorylated Bad separates from the apoptosis-promoting complex and forms a 14-3-3 protein complex, leading to the inactivation of its apoptosis-promoting function, and inhibits apoptosis [50]. Our results show that gypenoside effectively regulates the expression of apoptotic PI3K/Akt/Bad pathway-related proteins, and gypenoside could enhance PI3K and p-Akt expression and downregulate expression of p-Bad, Cyt-c, cleaved caspase 3, cleaved caspase 9, and PARP. These results suggested that gypenosides may regulate mitochondrial function and inhibit the development of atherosclerosis in ApoE−/− mice through the PI3K/Akt/Bad pathway.

The processes of mitochondrial fusion and cleavage are the main conditions affecting mitochondrial function. Dynamin-related protein 1 (DRP-1) is an important protein of the mitochondrial fission machinery and is involved in inducing mitochondrial fragmentation/degradation and programmed cell death [51]. Overexpression of the essential protein Drp-1 can promote mitochondrial division [52], promoting reactive oxygen species (ROS) and release of Cyt-c [53]. Mitochondrial fusions (Mitofusins) are a group of large GTPase localized on the outer membrane of mitochondrial [54]. The depletion of Mitofusin 2 (Mfn2) could greatly decrease levels of mitochondrial fusion [54]. Additionally, Mfn2 can increase the permeability of the extracellular membrane and induce Cyt-c release by inhibiting the PI3K/Akt pathway, promoting apoptosis [55]. In this study, we found that gypenoside significantly downregulated (*P* < 0.01) DRP1 and MFN2 protein levels. Thus, mitochondrial fusion cleavage proteins may promote the development of atherosclerosis in ApoE−/− mice via inhibiting the PI3K/Akt/Bad signaling pathway and regulating apoptosis in vascular endothelial cells. These results also demonstrated that the mitochondrial fusion cleavage proteins were significantly inhibited, suggesting that the total glucosides of Gynostemma pentaphyllum regulate mitochondrial function and inhibit the formation of atherosclerosis in ApoE−/− mice via the PI3K/Akt/Bad pathway.

Gypenosides may also restrain the formation of atherosclerosis through compensating for mitochondrial apoptosis and energy changes through compensatory stress mechanisms [15]. Our study showed increased expression of mitochondrial apoptosis-related proteins in the arterial endothelial cells of Apo E−/− mice, and the mitochondrial energy-related genes Atp12a, Cox5a, Ndufb6, and SDHC mRNA were also significantly elevated. After intervention with gypenosides, these results were significantly reduced.

In conclusion, this study identified a new effect of gypenosides on endothelial apoptosis and demonstrated gypenoside may be a therapeutic drug of atherosclerosis by modulating mitochondrial function through the PI3K/Akt/Bad pathway.

## Figures and Tables

**Table 1:** Primers used for quantitative real-time PCR.

Gene	Sequence	Product size
Sdhc	5′-TTGTATCAGAAAATGGTCTCTTCCT-3′ 5′-ACAGCCAGACCTGGGGTATT-3′	120 bp
Ndufb6	5′-GGAGCTAAGGAGACGATGGC-3′ 5′-TGGTTTAGTCATGTTCTTCCACA-3′	113 bp
Cox5a	5′-TGTCTGTTCCATTCGCTGCT-3′ 5′-TGACAGTCACCAACTCACACA-3′	107 bp
Atp12a	5′-GCACCATCATGATCAACGGC-3′ 5'-GACAGAAACCCAACACACGC-3′	118 bp

**Table 2:** Differentially regulated mitochondrial energy-related gene expression in the mouse aorta.

Gene	Description	GeneBank accession number	Fold change
Atp5g1	ATP synthase, H+ transporting, mitochondrial F0 complex, subunit c1 (subunit 9)	NM_007506	9.1172
Atp12a	ATPase, H+/K+ transporting, nongastric, alpha polypeptide	NM_138652	5.5417
Cox5a	Cytochrome c oxidase, subunit Va	NM_007747	10.5034
Cox6c	Cytochrome c oxidase, subunit VIc	NM_053071	6.7845
Cox7b	Cytochrome c oxidase subunit VIIb	NM_025379	6.6125
Ndufa4	NADH dehydrogenase (ubiquinone) 1 alpha subcomplex, 4	NM_010886	5.4346
Ndufa5	NADH dehydrogenase (ubiquinone) 1 alpha subcomplex, 5	NM_026614	4.0653
Ndufb6	NADH dehydrogenase (ubiquinone) 1 beta subcomplex, 6	NM_001033305	6.3204
Ndufb7	NADH dehydrogenase (ubiquinone) 1 beta subcomplex, 7	NM_025843	6.119
Ndufc2	NADH dehydrogenase (ubiquinone) 1, subcomplex unknown, 2	NM_024220	6.0068
Ndufs2	NADH dehydrogenase (ubiquinone) Fe-S protein 2	NM_153064	4.0314
Ndufv1	NADH dehydrogenase (ubiquinone) flavoprotein 1	NM_133666	4.4053
Sdhc	Succinate dehydrogenase complex, subunit C, integral membrane protein	NM_025321	7.8206
Uqcrc2	Ubiquinol cytochrome c reductase core protein 2	NM_025899	7.9425
Uqcrfs1	Ubiquinol cytochrome c reductase, Rieske iron-sulfur polypeptide 1	NM_025710	4.1404

## Data Availability

The datasets used and analyzed during the current study are available from the corresponding author upon reasonable request.
